# Development and application of a scoring system for septum injuries in beef calves with and without a nose flap

**DOI:** 10.1093/tas/txad075

**Published:** 2023-07-07

**Authors:** A A Kirk, C B Tucker

**Affiliations:** Center for Animal Welfare, Department of Animal Science, University of California, Davis, CA 95616, USA; Center for Animal Welfare, Department of Animal Science, University of California, Davis, CA 95616, USA

**Keywords:** beef, calf, injury, nose flap, two-stage, wean

## Abstract

The weaning period is a stressful time for beef calves because they must quickly gain independence from their dam. Gradual methods of weaning, such as when the calf is fitted with a nose flap to prevent suckling, are known to reduce the behavioral and physiological indicators of stress. Nose flaps are held in place by the nasal septum and are worn for 4 to 7 d. In the present study, the objectives were to 1) identify if a plastic nose flap worn for 7 d caused nasal injuries, (2) identify if factors like calf body weight or septum size predict injuries or flap loss, and (3) create a scoring system that could reliably score wound characteristics. Eighty-two (*N* = 82) Angus and Angus–Hereford crossbred beef calves were randomly assigned to ‘Flap’ or ‘No Flap’ treatments. Calves weighed 247 ± 29 kg and those with a flap had septums that were 39 ± 2 mm (mean ± SD). Images were taken of each nostril before flap insertion, on the day of removal, and 6 d after removal. Wounds were scored for the presence/absence of three characteristics in either nostril: damage (tissue where the flap rested was a different color than surrounding nostril), impression (edges of the wound were clearly raised or sunken), and blood. One trained observer scored a subset of photos (*N* = 64) twice, in a consistent manner for all three characteristics (damage, impression, and blood; 97%, 91%, and 100% agreement between 1st and 2nd evaluations, respectively), indicating that our system is repeatable. Thirty-two percent of calves in the Flap treatment lost their flap before the day of removal. No calves in the No Flap treatment were injured. All animals that kept their flap in for 7 d had damage and impressions in at least one nostril and 86% of calves had blood present immediately after nose flap removal (*P *≤ 0.001 compared to No Flap) indicating that the flaps altered the nasal tissue and created open wounds. Six d after flap removal, 100% still had visible damage, 64% had impressions, and 29% had blood, indicating that while damage is longer lasting, wounds can start to repair after the flap is removed. Injuries were prevalent in all calves, thus there was no relationship between calf size (body weight or septum width) on these wounds (*P* ≥ 0.374). Body weight or septum size did not differ (*P* ≥ 0.489) between calves that kept or lost their flap. Injuries inflicted from a nose flap may counteract the previously documented benefits of this method of weaning, making it less advantageous than alternatives and raise concerns about other uses of these devices in other contexts.

## Introduction

The weaning period is a particularly stressful time for beef calves because they must quickly establish nutritional and social independence from their dam (Weary et al., 2008). In the United States, beef calves are typically weaned from their dams between 4 to 8 mo of age (USDA, 2020). At this time, calves may be abruptly separated and experience changes in their diet, social structure, and their environment all at once. When separated, abruptly weaned calves responded with more behaviors that are indicative of distress like vocalization and walking compared to calves who underwent a gradual weaning method (Price et al., 2003; Haley et al., 2005). Calves who were abruptly weaned also gained less weight after separation compared to calves when the process was gradual (Price et al., 2003; Taylor et al., 2020). Biomarkers indicative of stress, such as increased blood cortisol or noradrenaline were markedly greater in abruptly weaned calves compared to gradually weaned counterparts (Hickey et al., 2003; de la Cruz-Cruz et al., 2021). In addition, an altered immune response and neutrophil concentrations was found in calves that were abruptly weaned compared to gradual methods (O’Loughlin et al., 2014; Lippolis et al., 2016; Lynch et al., 2019).

A purposed method to mitigate the distress that calves experience at weaning is the utilization of an antisuckling device, such as a nose flap. Developed in South America to temporarily prevent suckling in 2 to 3 mo-old calves (Hötzel et al., 2012), nose flaps are used in North America to gradually wean older beef calves in two stages. A plastic or metal flap is inserted into the nose and is held in place by the calf’s septum in the first stage (Haley et al., 2005, Lambertz et al., 2015; Taylor et al., 2020). This provides a physical barrier that prevents the calf from suckling while maternal–offspring contact is maintained to simulate a more naturalistic weaning process (Haley et al., 2005; Weary et al., 2008). After a given time period, the second stage involves the removal of flap and then physical separation from their dam. Calves weaned with a nose flap demonstrated fewer behaviors indicative of distress such as vocalization and locomotion than abruptly weaned calves (Haley et al., 2005; Loberg et al., 2008; Lambertz et al., 2015). When compared to abrupt weaning, calf weight gain was not affected by the nose flap (Burke et al., 2009; Lambertz et al., 2015; Alvez et al., 2016). Some researchers have reported that calves had a decrease in daily weight gain when the nose flap was in place, but similar overall gains compared to abruptly weaned calves (Haley et al., 2005; Burke et al., 2009; Valente et al., 2022). Plasma cortisol concentrations remained in normal range 0.3 μg/dL (Hopster et al., 1999) to 5.5 μg/dL (Doornenbal et al., 1988) for calves that were weaned with a nose flap (Freeman et al., 2021) suggesting that this method did not cause additional stress to the calves. Overall, there are behavioral advantages to nose flap weaning, and even if the physiological indicators may be initially negative calves are expected to recover from the stress relatively quickly.

Although weaning with a nose flap is generally seen as having net gains for animal welfare, there have been concerns about injuries inflicted on the nasal septum during the process. Modest to moderate nasal injuries (Lambertz et al., 2015; Taylor et al., 2020), hemorrhage, ulceration, erosions (Taylor et al., 2020), abrasions (Lambertz et al., 2015), lesions (Freeman et al., 2021), or open wounds (Valente et al., 2022) have been observed at the time of flap removal. Although uncommon, more serious, or life-threatening abscesses (Fernandes et al., 2000; Loretti et al., 2003) have also been reportedly caused by wearing of plastic nose flaps.

To date, nose flap induced injuries have only been characterized in two studies. Lambertz et al. (2015) and Valente et al. (2022) both developed scoring systems that assigned a score or grade to the injury to classify its severity. Lambertz et al. (2015) evaluated nasal abrasions associated with the use of plastic and metal nose flaps held in place for 7 d and observed nasal abrasions in over 95% of calves at flap removal. Of these animals, 27% had slight irritation, 58% of those had slight or heavy bleeding, about 7% had pus, and 4% had deep purulent wounds (Lambertz et al., 2015). Similarly, Valente et al. (2022) identified presence of injury and different types of secretion; including blood, and translucent or purulent secretion, and found that all calves (41 of 41) all fell into one of the ‘injured with or without secretion’ categories at flap removal. Both studies had mutually exclusive descriptions for wounds, scored live, and did not have controls where calves were kept in the same environmental conditions to ensure that injuries were solely caused by the nose flap. All these factors may have affected external validity and repeatability of the scoring systems.

Multiple types of nose flaps are commercially available (Supplemental Table S1, https://doi.org/10.5281/zenodo.7349134; Kirk and Tucker, 2023) and have been studied in a range of calf ages and sizes (Supplemental Table S2, https://doi.org/10.5281/zenodo.7349134; Kirk and Tucker, 2023). Throughout these different designs and dimensions, the goal of the nose flap is the same: to stay in place while the calf is being weaned. To do this, the flap must have a narrow gap to hold it in place, but it is common for these methodological details to be omitted in the description of the flap design. The closest information in terms of the functional aspects of attachment to the nasal septum is how many flaps were lost before removal. While Haley et al. (2005) and Taylor et al. (2020) both reported less than 5% flap loss, others have reported 24% (Lambertz et al., 2015) or even 27% (Valente et al., 2022) loss of the flap before calves had them manually removed. At the time of the current study, nose flap loss has not been evaluated in relationship to calf size or nostril septum width measurement.

The present study investigated nasal injuries inflicted by nose flaps and examined the relationship between calf weight or nasal septum size and these wounds. The objective was to develop a reliable scoring system to characterize any wounds observed. It was hypothesized that heavier calves or those with wider nasal septum measurements would have more injuries inflicted by the nose flap.

## Materials and Methods

### Animals and Housing

All procedures involving animals were approved by the University of California-Davis Institutional Animal Care and Use Committee (Protocol #22254). This study was conducted at the Sierra Foothill Research and Extension Center (Browns Valley, CA) facility from May through June 2021. Eighty-two (*N* = 82) Angus and Angus–Hereford crossbred beef heifer (*N* = 37) and steer (*N* = 45) calves were utilized. Sample size was determined based on availability of calves and feasibility of restraint and photography in the time we had allotted for this study. The mean age of calves enrolled in this study was 205 ± 19 d old and weighed 247 ± 29 kg, ranging from 186 to 306 kg (mean ± SD, min to max values). Regardless of treatment, all calves were weaned with the fence line method, where they were placed in an adjacent pasture that separated them from their dams with a fence, as part of another project. On the day of weaning, calves were handled in a squeeze chute, weighed, and flaps were inserted. Calves were housed on California rangeland pasture for the entirety of the study, all under the same conditions with ad libitum access to water. This approach allowed us to equalize the environmental risk of injury from nonflap sources.

Data (https://doi.org/10.25338/B8535Z), RMarkdown file (https://doi.org/10.5281/zenodo.7349132), and Supplementary Materials (https://doi.org/10.5281/zenodo.7349134) are available online in the Dryad repository (Kirk and Tucker, 2023).

### Experimental Design and Treatments

Calves (*N* = 82) were randomly assigned to one of two treatments, balanced for body weight, and checked that calf sex was balanced between treatments. On the day of flap insertion, all calves were gathered, restrained in a squeeze chute, then vaccinated, and weighed. Images of each nostril were taken before flap insertion with a Nikon D5300 camera with 18 to 55 mm NIKKOR VR II lens kit attachment (Nikon Inc. Melville, NY). A ring light, ProMaster RL100 Macro LED Ring Flash (ProMaster, Fairfield, CT) was used to illuminate and provide consistent lighting. Images were taken of the left and right inner nostril and focused on the septum for all animals; all images were taken approximately 30 cm away. Then, calves who received the nose flap treatment (*N* = 41) had plastic nose flaps (Quiet Wean; JDA Livestock Innovations, Ltd, Saskatoon, Canada) inserted.

All calves were gathered 7 d after flap insertion (FLAP REMOVAL). The calves who received nose flaps and kept them for the entire 7 d (*N* = 28) had them removed. These calves were noted as FLAP ENTIRE. Calves who lost the flap before the 7 d period ended (*N* = 13 or 32%) were noted as FLAP PARTIAL. Images of left and right inner nostrils were taken at flap removal with the same photography procedure for all treatments.

A third set of images were taken a further 6 d after flap removal (6 D AFTER) for all calves that received nose flaps (FLAP ENTIRE and FLAP PARTIAL). Additionally, septum measurements were taken with a 150 mm MC1630EWRI Digital Caliper (Mahr GmbH, Göttingen, Germany). Measurements were the distance across the nose, between the nostrils as a proxy for the septum width.

Images were assessed and scored by a single trained observer using the nasal injury definitions outlined in Table 1. The observer scored a subset of photos (*N* = 64 nostrils or 32 calves) twice, in a consistent manner for all three characteristics (damage, impression, and blood, 97%, 91%, and 100% agreement between first and second evaluations, respectively). Then this observer (AAK) scored the photos from all three time points (before insertion, at removal, 6 d after removal). Images taken after removal of the nose flaps were compared to the same animal before nose flap insertion to identify natural color variation in the nostril. If the injury was occluded by dirt, the image was scored “NA”. The observer was not blinded to the treatments.

**Table 1.** List of features scored, definitions and examples of nose flap injuries in weaned beef calves. Features are not mutually exclusive

Feature	Definition	Example
No injury	Absence of all injury characteristics	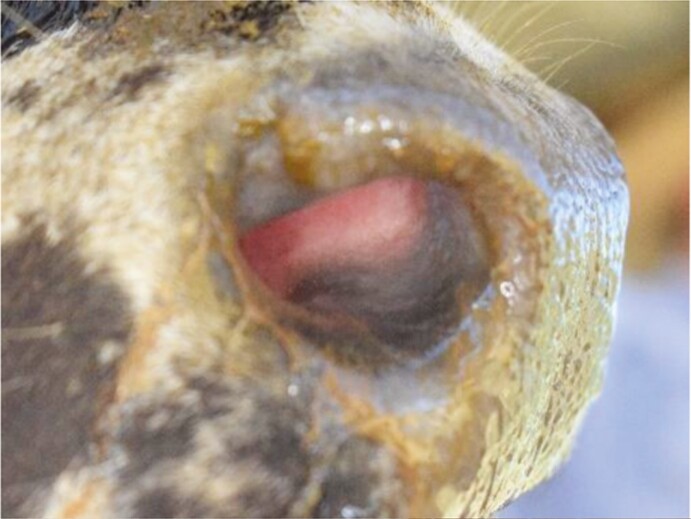
Evidence of damage (yes/no)	The tissue in the site where the flap would rest is a different color than surrounding nostril; natural variation in nostril color^1^ (e.g., spots) are not counted as wound-related difference	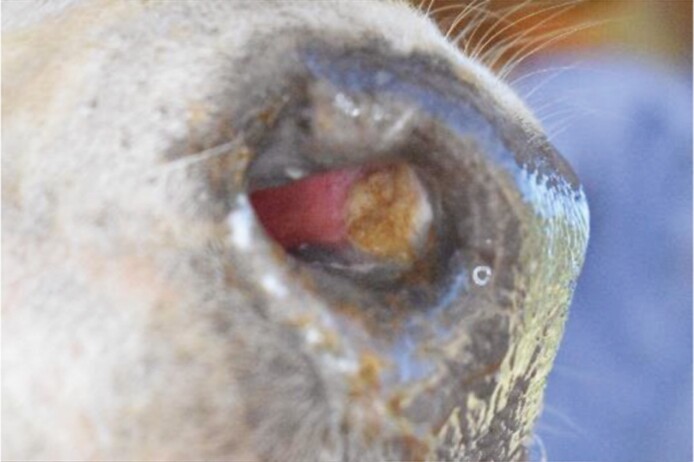
Visible impression (yes/no)	Edges of the wound are clearly raised or sunken, distinct from surrounding tissue. May be an entire or partial circle or oval	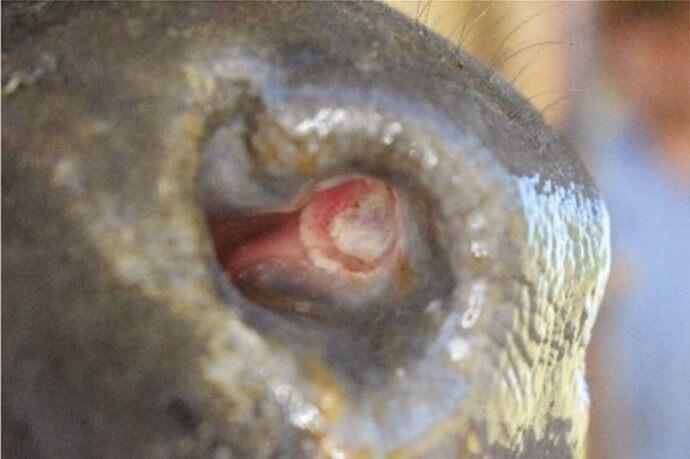
Visible blood (yes/no)	Bright red liquid present in the nostril area, either in or around the site where the flap would rest	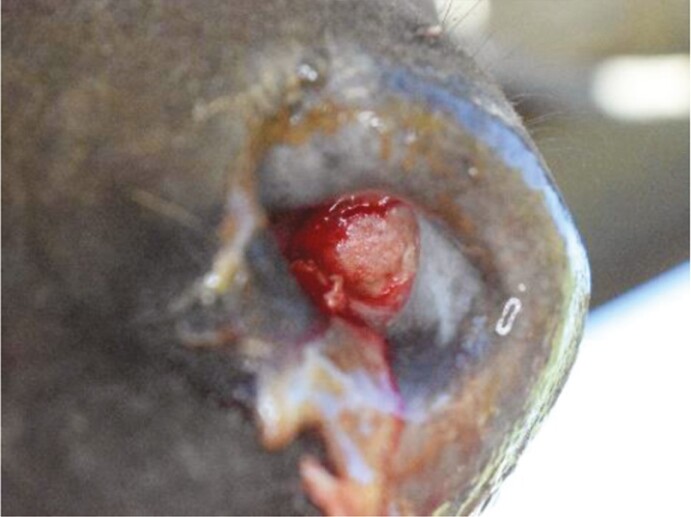

^1^As assessed in photos before flaps were inserted.

### Statistical Analysis

Initially, 82 calves were included in this study and half (*N* = 41) received the flap treatment. The 13 FLAP PARTIAL calves were only included in data analysis comparing them to FLAP ENTIRE. Two of the calves in the NO FLAP treatment were excluded due to being missed during a handling event. A total of three characteristics (one damage and two impression) were scored “NA” due to a blurry photo or dirt in the wound making it difficult to score.

Injury data were entered in Microsoft Excel (Redmond, WA; 2016) and it was noted if each calf had a given characteristic in at least one nostril. Analyses were conducted in R version 4.1.2 (R Core Team, 2021) via R Studio version 2021.09.02 (R Core Team, 2021). Presence of impression, damage, and blood in at least one nostril for FLAP ENTIRE and NO FLAP calves was analyzed with separate Fisher’s exact tests for each type of injury in the scoring system (*fisher.test* function, stats package version 4.1.2). Relationships between each injury characteristic and body weight, as well as each characteristic and septum size, were analyzed with a binomial regression (*glm* function, dplyr package version 1.0.9; Wickham et al., 2022) with weight and septum size specified as a fixed effect and family entered as binomial (link = “logit”). Assumptions were checked with a variance test (*var.test* function, base R version 4.1.2) and Shapiro–Wilk test (*shapiro.test* function, dplyr package version 1.0.9; Wickham et al., 2022). Data were also visualized with QQ plots (*qqnorm* and *qqline* functions, stats package version 4.1.2). The comparison of body weight between FLAP ENTIRE and FLAP PARTIAL calves was done with a Wilcoxon Signed Rank Test (*wilcox.test* function, base R version 4.1.2) because the assumption of normality was not met. The comparison of septum size was done with a two-sample *T*-test (*t.test* function, base R version 4.1.2) with equal variance specified.

## Results

No calves (0 of 39) in the ‘No Flap’ treatment had damage, impression, or blood present on day 7. In contrast, all calves that kept their flap in for 7 d had at least one nostril with damage (28 of 28) and impression (28 of 28), and 86% (24 of 28) had blood present in at least one nostril immediately after flap removal (*P *≤ 0.001 compared to No Flap) for each characteristic (Table 2). A description of whether an injury characteristic occurred in one, or both nostrils is outlined in Table 3.

**Table 2.** Percent of animals with damage, impression, or blood present in calves that received a nose flap and kept it for the entire 7 d (FLAP ENTIRE) or did not receive a nose flap (NO FLAP). Data were collected before the flaps were inserted, at flap removal and 6 d after flap removal

	Damage		Impression		Blood	
	FLAP ENTIRE	NO FLAP	FLAP ENTIRE	NO FLAP	FLAP ENTIRE	NO FLAP
Before flap	0	0	0	0	0	0
Flap removal	100	0	100	0	86	0
6 d after removal	100	N/A	64	N/A	28	N/A

**Table 3.** Percent of animals with damage, impression, or blood present in one or both nostrils. (FLAP ENTIRE). Data were collected at flap removal and 6 d after flap removal

	Damage		Impression		Blood	
	ONE	BOTH	ONE	BOTH	ONE	BOTH
Flap removal	1/28	27/28	1/28	27/28	5/24	19/24
6 d after removal	0/28	28/28	11/18	7/18	8/8	0/8

Wounds were scored 6 d after flap removal and 100% (28 of 28) of calves had at least one nostril that still had visible damage, 64% (18 of 28) of calves had impressions, and 29% (8 of 28) had blood present.

**Figure 1.**
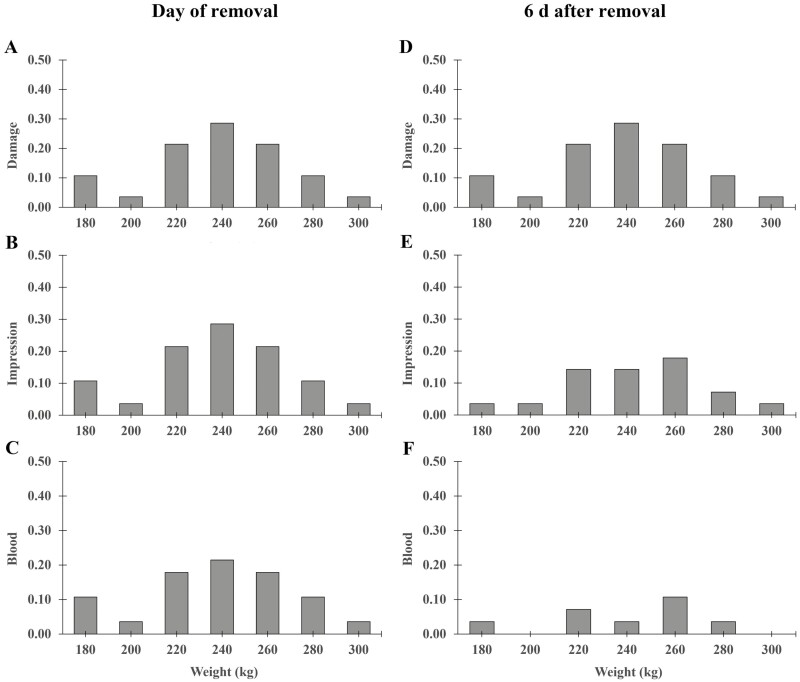
Proportion of FLAP ENTIRE (*N* = 28) calves with at least one nostril with nasal wound characteristics (A) Damage, (B) Impression and (C) Blood, in relationship to the animal’s body weight (in kg) on the day of removal and 6 d afterward (D, E, F). There were no animals with damage, impressions, or blood present in the NO FLAP treatment.

**Figure 2.**
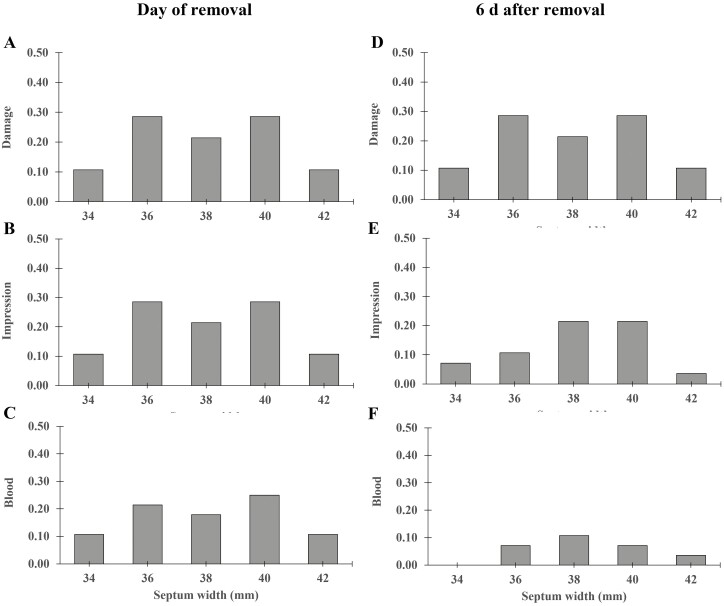
Proportion of FLAP ENTIRE (*N* = 28) calves with at least one nostril with nasal wound characteristics (A) Damage, (B) Impression and (C) Blood, in relationship to the animal’s septum width (in mm) on the day of removal and 6 d afterward (D, E, F). There were no animals with damage, impressions, or blood present in the NO FLAP treatment.

Body weight did not have an effect on damage, impression, or blood presence on the day of removal (*P *≥ 0.824) or 6 d afterwards (*P* ≥ 0.632; Figure 1). Similarly, septum width did not have an effect on damage, impression, or blood on the day of flap removal (*P* ≥ 0.797) or 6 d afterwards (*P* ≥ 0.374; Figure 2).

Calf body weight or septum width did not have an effect on flap loss (Supplemental Table S3, https://doi.org/10.5281/zenodo.7349134; Kirk and Tucker, 2023). Calves that kept their flap for the entire 7 d weighed 249 ± 6 kg and those that lost their flap prior to removal weighed 242 ± 7 kg (mean ± SE; *P = *0.393). Similarly, septums of calves that kept the flap measured 39 ± 0.4 mm and those that lost it were 39 ± 0.6 mm (mean ± SE; *P* = 0.464).

## Discussion

The present study investigated if nose flaps that were worn by ~7-mo-old beef calves for 7 d caused nasal injuries. Injury characteristics (damage, impression, or blood) were absent in calves that did not receive a nose flap. However, a high occurrence of nasal injuries in all calves who retained the flap for 7 d both at flap removal and 6 d afterwards was observed. Additionally, nasal injuries were present across all body weights and septum widths, therefore, these factors did not have an effect on nasal injuries within the range of sizes in the current study. Lastly, it was demonstrated that these wounds could be scored in a repeatable and consistent manner.

Although nose flaps used in calves has been regarded as a weaning method that can reduce behavioral signs of distress like vocalization and walking when compared to abruptly separated calves (Haley et al., 2005; Enríquez et al., 2011; Alvez et al., 2016; Freeman et al., 2021); the incidence of nasal injury should be considered because it is a potential drawback to using this method. Flaps can also be used to temporarily stop suckling for reproductive management in dams (Orihuela and Galina, 2019; Santa Cruz et al., 2022), or may be used to address cross- or inter-sucking in dairy breeds (Keil et al., 2000; Lidfors and Isberg, 2003). While several studies with flaps simply mentioned nasal injuries, Lambertz et al. (2015) and Valente et al. (2022) developed scoring systems to score or grade to the injury based on its severity. Lambetz et al. (2015) used an ordinal scale with scores 0 through 6. A score 0 meant no irritations while 6 indicated a perforated septum or fatal wound (Lambertz et al., 2015). Valente et al. (2022) identified presence of injury and different types of secretion; including blood, and translucent, or purulent secretion. While these were instrumental in describing the incidence and merit of wounds, these scoring systems had mutually exclusive descriptions of each wound characteristic, were scored live, and lacked a no flap control in the same environment. In contrast, the current study captured high-definition images of nasal injuries and scored these for absence or presence of each wound characteristic, where each component was scored on its own right, and were not mutually exclusive. Authors in the current study also ensured that the images were reliably scored in duplicate to determine if this scoring system was repeatable over all time points. This approach also considered the natural variation of nostril color with our photos from before the flap was inserted. Additionally, No Flap calves acted as a control and allowed to rule out any external environmental factors that may have caused nasal injuries.

In this population of ~7-mo-old calves, a 100% prevalence of nasal damage at removal and 6 d after removal was observed in calves that received a nasal flap. This characteristic included any alteration caused by the nose flap that damaged the epithelial cells of the septum skin barrier. Inner-nasal lining is made of highly vascularized tissue that is covered by a continuous layer of mucous (Harkema et al., 2006). Damage to this tissue may be caused by pressure, shear, friction, moisture, or a combination of these factors (Kottner et al., 2009) and sustained inward pressure on the nasal septum by the nose flap. Valente et al. (2022) also described abrasions caused by the rubbing of the nose flap against the skin of the inner septum. In the present study, no perforated nasal septums were observed, but substantial injuries to the superficial layer of the nasal septum, like those reported by Valente et al. (2022) was reported. Because this characteristic was still prevalent 6 d after flap removal (100%), it suggests that damage is longer lasting, but it is unknown how long they take to resolve.

Impressions were present at removal (100%) and became less common 6 d afterwards (64%). This characteristic described the edges of the wound as being slightly raised or sunken compared to the surrounding nostril tissues. It was noticed that the flap made a circular shaped indentation into the nasal tissues where it rested, similar to the wounds pictured in Valente et al., (2022). Bisang et al. (2022) also mentioned round superficial ulceration upon nose ring removal in dairy calves. General wound healing progression includes three stages: inflammation, new tissue formation, and remodeling; remodeling involves the proliferation of cells from the edges of the wound to restore the protective barrier (Watelet et al., 2002; Gurtner et al., 2008). After the flap was removed, the nasal mucosa may have been proliferating cells along the edge of the damaged tissue as a result of inflammation, or an attempt to form new undamaged tissues. This may have also caused impression to vary in one or both nostrils.

Presence of blood was observed in 85% of calves on the day of flap removal and was reduced to 28% 6 d afterward, suggesting that there were fewer open wounds or ruptured blood vessels at that time. Results from the current study resembled findings from Lambertz et al. (2015) when approximately 58% of calves that received nose flaps had slight or heavy bleeding at the time of flap removal, and only 30% had slight or heavy bleeding when observed 1 wk later. There was markedly more bleeding in the present study than Valente et al. (7% 5 d later at removal; 2022) and Freeman et al. (32% at removal; 2021).

The fit of the nose flap appears to be an important consideration for this method. The flap must fit securely to the calf’s septum, so it will not fall out before the herd managers remove it. If flap loss does occur, the calves could resume suckling, and advantages of a gradual weaning process would be lost. In the present study, there was a 32% (13 of 41) loss of the flaps before day 7. These calves were excluded in the analysis because they were housed on rangeland pasture, so it was difficult to determine how or when these calves removed them. These findings contrast with Haley et al. (2005), who reported less than 5% of calves lost their flap over four trials, and Taylor et al. (2020) who reported losing one flap before the day of removal, but the prevalence of injury in many calves. The results from our study were better aligned with Lambertz et al. (2015) and Valente et al. (2022) who reported a 24% and 27% loss, respectively. It is unclear why the current study had the highest percent of flap loss, because calf size, average 247 kg, was similar to the previous studies and the national average for the United States (USDA, 2020). Flap type, calf nose size, and inexperience of fitting the nose flaps to the calves may have played a role in this. Future research could include observing calves for attempts to remove the nose flap or behaviors like head shaking to identify if flaps are uncomfortable. Additionally, calves kept with their dams would be motivated to nurse, creating potential rubbing or friction induced injuries that may warrant research.

In this study, calves wore the nose flap for 7 d. Manufacturer recommendations indicate that flaps should be kept in for 4 to 7 d before removing them and separating the calf from the cow (QuietWean, 2021). Previous studies (Lambertz et al., 2015; Freeman et al., 2021) have also kept the flap fitted to the nasal septum for 7 d and mentioned nasal injuries. However, some studies describe the occurrence of nasal injuries when the flap was fitted for only 5 d (Valente et al., 2022) or 6 d (Taylor et al., 2020). Although not recommended, periods longer than 7 d have also been evaluated such as 14 d (Loberg et al., 2008), 17 d (Enríquez et al., 2010), or 21 d (Alvez et al., 2016; Lippolis et al., 2016) to align with other aspects of weaning procedures, such as vaccination schedules. Based on our results, we would not recommend keeping the flap in for longer time periods.

## Conclusions

In conclusion, this study contributed to our knowledge about gradual weaning methods and use of nose flaps. Our results suggest that all ~7-mo-old calves who kept a nose flap in for the entire 7 d had injuries, and these persist at least 6 d after removal. We developed a reliable scoring system to describe characteristics visible in these injuries. Calf weight or septum size did not predict the occurrence of injury characteristics or flap loss. While nose flap weaning conveys animal welfare benefits over abrupt weaning, incidence of nasal injury should be considered because it is a potential drawback to this method and uses of these devices in other contexts.
